# ‘Hard-pressed on every side’: Christian women’s experiences of intimate partner violence in two provinces of South Africa

**DOI:** 10.4102/hsag.v28i0.2333

**Published:** 2023-11-06

**Authors:** Tshilidzi R. Nevhutanda, Mahlasela A. Rakhudu, Lufuno Makhado

**Affiliations:** 1 Department of Nursing Sciences, Faculty of Health Sciences, North-West University, Potchefstroom, South Africa; 2 Department of Public Health, Faculty of Health Sciences, University of Venda, Thohoyandou, South Africa

**Keywords:** Christian women, intimate partner violence, IPV survivors, lived experiences, Pentecostal churches

## Abstract

**Background:**

Women in South Africa are battling the scourge of intimate partner violence (IPV), and men are usually responsible. Despite this, no studies have been carried out to specifically explore and describe Christian women’s IPV first-hand experiences in the Limpopo and Gauteng provinces.

**Aim:**

This study explored and described lived experiences with regard to IPV among women attending Pentecostal churches in Limpopo and Gauteng provinces.

**Setting:**

The study was conducted in the Makhado Local Municipality, City of Tshwane and Johannesburg metropolitan municipalities.

**Methods:**

The study utilised a qualitative research method, using interpretative phenomenological analysis. Sampling was purposeful. Semi-structured individual interviews were conducted to collect data among women attending Pentecostal churches in Limpopo and Gauteng provinces. Data saturation was achieved after 10 survivors were interviewed.

**Results:**

Two themes surfaced from the analysis: the experience of various forms of IPV by survivors and the hardships survivors encountered when seeking help after IPV.

**Conclusion:**

In spite of the salient role Christian belief played in survivors’ lives, the expected support from family, pastors and law enforcement officers after disclosing IPV was non-existent. The survivors were stranded, frustrated and displayed symptoms of depression and anxiety.

**Contribution:**

This study revealed survivors’ need for comprehensive, multidisciplinary, and transdisciplinary collaborative support by strengthening partnerships with church leaders to protect Christian women against IPV.

## Introduction

South Africa takes fourth place globally in terms of excessive acts of gendered violence (Yesufu 2022:96), and this is an extremely concerning position the country finds itself in. Of the various types of gender-based violence (GBV), intimate partner violence (IPV) is a considerably rampant type of GBV (Nguyen et al. 2022:2), and has been found to affect 20% to 50% of women in South Africa (Sere, Roman & Ruiter 2021:2). The high levels of underreporting make it difficult to determine the exact figures.

Violence is perceived differently within different contexts, making it difficult to define. Physical violence, for example, can be justified as self-defence. A variety of factors have been found to cause or contribute to violence, and these include individual factors such as low educational levels that lead to an acceptance of abuse, relational factors such as financial dependence on an abusive and controlling partner, and socio-cultural factors such as patriarchy that exacerbates existing inequalities (Apiribu, Duma & Ncama 2020:15). According to Westenberg (2017:1), one notable factor contributing to violence against women is the environment, and churches are one such environment that potentially perpetuates and conceals IPV. Rather than the current miscue of scriptures, Shaw et al. (2022:979) encourage churches to offer appropriate support to victims and urge church leaders to address the way the concept of submission within Christian marriages has been misconstrued and has led to the perpetuation of violence against women.

Authors including Asay et al. (2016), Westenberg (2017:8), Malesa (2021:156); Mkhize, Khanyisile and Olofinbiyi (2022:8); Khosa-Nkatini and Mofokeng (2023:5) have documented the prevalence of IPV within Christian homes and faith communities, as well as the manner in which the church has not offered appropriate support to victims of IPV.

Although the abuse may be known, it is not openly discussed. According to Banda (2020:2), the church tends to react to the loss of life because of abuse rather than proactively addressing prevention.

For the most part, churches across the board are struggling with issues of IPV, as IPV is considered a national pandemic affecting a variety of people across the population (churchgoer and nonchurchgoer). For example, other mission churches, such as Evangelical churches, are also said to display a meek approach to issues of violence within marriages, like their Pentecostal counterpart (Khosa-Nkatini & Mofokeng 2023:5). Furthermore, the authors posit that religious institutions might hold certain ideologies that potentially exacerbate marriage violence. For instance, the submission issue in many churches overlooks family dynamics and socio-economic factors. This outdated approach fails to recognise such factors and allows the church to shy away from issues of abuse within marriages (Khoza-Nkatini & Mofokeng 2023:5). Mkhize et al. (2022:8) also observe the biased nature of scripture used in the example of both wives and husbands submitting to one another and their bodies thereof, as this submission and ownership of bodies is seemingly only applicable to one gender. This action of forced sexual activity will exacerbate marital rape.

Malesa (2021:156) believes that the church can be influential in reforming the attitudes men hold towards women by upholding a higher esteem of women within marriages, which should, in turn, make them less vulnerable to abuse. The church is not seen as a reliable conciliator when it concerns abuse within marriages. Despite this, fewer studies conducted in South Africa document the lived experiences of women experiencing IPV within Pentecostal churches.

### Problem statement

Many researchers agree that IPV is a problem. The problem noticed by Jung and Olson (2017:609) is that ‘the linkage between religiosity and IPV is complex, full of contradictions and ambiguities’. According to le Roux and Bowers-Du Toit (2017:22–23), religion contributes to the high levels of IPV in the country because the church chooses to remain tight-lipped about the matter.

It has been posited that churches legitimise abuse within marriage under the umbrella of marital sanctity by misinterpretation scriptures and prompting forbearance through prayer rather than acting against the abuse (Khosa-Nkatini & Mofokeng 2023:5; Le Roux & Bowers-Du Toit 2017:22–23). Likewise, the study participants shared these experiences as church leaders offered no substantial support but rather encouraged staying within their abusive marriages. This was immensely discouraging among the study participants, leaving them misguided and alone.

Damron and Jonson (2015:6) highlight the challenges women go through when scriptures are used as guidance by ill-informed church leaders. Kgatle and Spaumer (2023:2) question the validity and effectiveness of church leaders’ theological training and capabilities to address specific issues. Furthermore, perpetrators twist and misuse the same scriptures to control and oppress their partners. While studies on IPV experiences among women are extensive, fewer have documented the lived experiences of women attending Pentecostal churches, specifically in the Limpopo and Gauteng provinces. This motivated this study that investigated and described the abuse by intimate partners among Christian women in the abovementioned provinces.

### Research aim

This research explored the experiences of Pentecostal church women survivors of IPV and how they perceived church leaders’ counselling and support.

## Research methods and design

The study utilised a qualitative research method, employing an interpretative phenomenological analysis (IPA) to outline and expound on the spousal abuse of Pentecostal women in the provinces of Limpopo and Gauteng. The IPA design was the most suitable approach to provide a detailed exploration and description of the survivors’ lived experiences (Neubauer, Witkop & Varpio 2019:91).

### Study context

The study was conducted in the provinces of Limpopo and Gauteng. Limpopo is a rural province, with the majority of people (78.9%) living in poverty (Van der Walt 2020); in addition, the distribution of wealth is highly unequal. The 2021 population was estimated at just over 6 million people (Galaj 2022). The denominations in Limpopo are based in the Makhado Local Municipality of the Vhembe District, particularly in the Dzanani, and Makhado regions, with church members amounting to over 1500 (*n* = 1500) at the time of the study. Most of the Pentecostal churches in these two regions are not formally registered.

According to Galaj (2022), Gauteng is the smallest, most highly urbanised and most populous province, and the estimated population in 2021 was 15 488 137. In this Gauteng province, the research sites were in City of Tshwane and Johannesburg Metropolitan Municipalities. Research sites have a diverse representation of church leaders, with a combined total of 800 (*n* = 800) church members when data were collected.

### Population and sampling

The study population was selected based on the survivors’ experience of IPV and being members of the aforementioned churches. The IPV is rife in both Limpopo and Gauteng provinces. Meyiwa et al. (2017:8620) observes that almost 51% of women in Gauteng and 77% in Limpopo are exposed to IPV. The study’s population included married, divorced and remarried women within Pentecostal churches that have experienced IPV. Participant sampling was purposive and based on convenience. The researchers selected participants who were knowledgeable about the issue under study (Babbie 2017:196).

The researchers carefully checked aspects of the subject under study and communicated these to the pastors in both settings. The pastors in turn agreed to give leaflets which explained the purpose of the study, eligibility criteria and confidentiality issues, to the women in their churches. Twenty were married, divorced, and remarried women in the two provinces. These IPV survivors reached out to their leaders for guidance and assistance. Eleven participants who participated in the interviews were from Limpopo, although no additional data was found after the 5th interview. Similarly, in Gauteng, nine participants participated in the study, and no additional data was found after the 5th interview. The interviews were conducted at selected churches and places convenient for participants.

### Criteria for eligibility

The inclusion criteria of this study included any Christian woman married or divorced, experiencing or has experienced IPV, receiving counselling from their church leaders, and voluntarily agrees to participate in the study. Exclusion criteria were women who married or divorced but never sought counsel from church leaders, married or divorced and abused by other family members and strangers, and those who were never married and abused by partners or strangers. Some survivors wanted to be part of the sample but did not receive counselling from pastors because some of their abusers were either pastors or members of the church board, and they could not expose them. Others were of the view that counselling would not change their husbands’ abusive behaviours.

### Recruitment procedure and data collection

The recruitment procedure was done by posting flyers on noticeboards to inform participants about the research. The flyers had the researchers’ contact details. The researchers communicated to the pastors who, in both settings, agreed to give leaflets that had the purpose of the study, eligibility criteria and confidentiality issues to the women in their churches. The researchers followed Alase’s (2017:15) five-step procedure for recruitment and data collection. The first step in an IPA study is that the researcher should conduct semi-structured and unstructured interviews with between 2 and 25 participants. In this study, 10 participants were interviewed using ‘a semi-structured interview guide’ (Dziewa & Glowacz 2021:644). The researchers could dig into participants’ personal and sensitive information (DeJonckheere & Vaughn 2019:1–2). Participants were asked to describe their IPV lived experiences.

The second step in an IPA study is the duration of interviews, which is recommended to be 60–90 min. The duration of interviews in this study was between 60 min and 90 min. The third step in an IPA study is that one interview invitation per participant should be kept, but follow-up interviews can be performed if necessary. In this study, all the participants were interviewed once. The fourth step in an IPA requires that the participants decide the place, date and time. In this study, participants decided on the sites, dates and interview times. The fifth and final step in an IPA study was using technological devices, such as electronic voice recording devices, for data collection. In this study, the researcher used a voice recorder and a notepad to jot down the observations after obtaining participants’ permission for the use of a voice recorder. She introduced herself and explained their rights and what is expected of them in the interview.

The researchers interviewed participants individually and used the voice recorder to capture participants’ voices (De Vos et al. 2011:342), gathering information concerning survivors’ IPV lived experiences and getting a sense of how they perceived and viewed their counselling experiences.

### Data analysis

The researchers used IPA’s steps for data analysis as described by Smith and Osborn (2021:67–76). The first step involves extensive repeated reading and listening attentively to the recorded information to get clarity and to ensure that participants are the focus of the analysis (Smith & Osborn 2021:67). The second step is initial noting of the participants’ responses into meaningful phrases while developing emergent themes on the other margin. The researchers were able to re-organise the data written in the first step, turning the notes into relevant themes (Smith & Osborn 2021:69). The third step is searching for connectedness across emergent themes. The researchers then put these in chronological order, writing themes that emerged on the ‘right-hand margin’ according to their connection (Smith & Osborn 2021:70). The fourth step is clustering similar themes, ensuring that ‘the connections work for the primary source material – the actual words of the participant’ (Smith & Osborn 2021:72). The researcher presented the themes in the form of a table grouping them in two columns of themes and categories (Smith & Osborn2021:72). In the fifth step, the themes were translated in a manner that depicted the experiences of study participants (Smith & Osborn 2021:76).

An independent coder was sought for co-coding to ensure quality and validity. The co-coder was assigned the field notes and participant verbatim transcripts (Creswell & Poth 2018:212). The co-coder and researchers discussed the themes and categories within the data collected. This consensus discussion helped the types and themes of participants’ experiences (Miles, Huberman & Saldana 2013:117).

### Ethical considerations

The Health Science Research Ethics Committee of the North-West University (Ethics number NWU-00127-16-A9) provided the ethical clearance. Permission to approach Pentecostal churches was sought from associated church governing bodies in both the Limpopo and Gauteng provinces. The church leaders acted as gatekeepers, granted goodwill permissions and assisted in recruiting participants and negotiating informed consent. The researchers made sure that participants were willing to participate in the study voluntarily and sought written consent. She also took precautions to protect participants’ rights to privacy, anonymity and confidentiality. It was a priority for the researchers to respect participants who were assured that they could withdraw from participation voluntarily (Sebaeng, Davhana-Maselesele & Manyedi 2016:2). Participants gave informed consent. They were assured that withdrawing from participation is acceptable (Harker et al. 2022:5). A professional counsellor and a social worker were arranged in case the IPV survivors experienced emotional breakdowns during the interviews. The mediator, co-coder and social worker who participated in this study signed confidentiality agreements.

### Ensuring trustworthiness

Measures to safeguard the trustworthiness of research results were taken. Criteria used for ensuring trustworthiness were ‘credibility, transferability, dependability and confirmability’ (Botma et al. 2022:293–295). Credibility was achieved through prolonged engagement, persistent observation and member checking, where participants were asked to summarise their answers, and the researchers summarised and captured the information. The researchers achieved triangulation by using different data collection methods, including data from interviews and descriptive and reflexive field notes (Botma et al. 2022; Cope 2014:90). Authenticity was achieved by conveying participants’ true feelings and tone during the interviews (Botma et al. 2022:296). Peer review was achieved through co-coding, wherein an independent qualitative researcher was engaged (Botma et al. 2022:292). Conformability was achieved by providing rich quotes from participants (Cope 2014:89). Transferability was achieved through the purposeful selection of survivors of IPV and describing in detail participants or settings (Nieuwenhuis 2016:124) and comparing the sample with demographic information (Botma et al. 2022:295), ensuring that the results reflect the participants’ IPV experience. The counselling experience of the researchers was bracketed to avoid intimidating participants while collecting sensitive information.

## Results

### Demographic information of participants

Ten IPV survivors participated in the study, represented as follows: Pentecostal Church 1 Gauteng and Pentecostal Church 2 Limpopo. The participants were aged between 30 and 55 years old and had endured abuse over a period ranging from 3 to 30 years. The survivors revealed experiences of IPV, various types of violence and challenges they experienced when seeking help. The participants’ demographics were used with their words to elaborate on study results. Two main themes and eight categories were identified. Data analysis produced the findings, as outlined in Table 1 and Table 2.

**TABLE 1:** Demographic information.

Participant code	Province	Pentecostal church number	Age	Occupation	Relationship (Years)
F1	Gauteng	1	30	Manager	5
F2	Gauteng	1	35	Manager	9
F3	Gauteng	1	35	Quality assurer	3
F4	Gauteng	1	38	Senior manager	9
F5	Gauteng	1	34	Accountant	7
F6	Limpopo	2	36	Self-employed	8
F7	Limpopo	2	54	Lecturer	27
F8	Limpopo	2	37	Housewife	12
F9	Limpopo	2	55	Senior lecturer	30
F10	Limpopo	2	45	Clerk	10

**TABLE 2:** Summary of findings.

Theme	Category
Survivors revealed experiences of various forms of IPV	Psychological violence
	Physical violence
	Financial abuse
	Sexual violence
	Social isolation
Survivors encountered hardships while looking for assistance after IPV	Difficulties when disclosing partner abuse to their family
	Difficulties when disclosing partner abuse to their church leaders
	Difficulties when disclosing partner abuse to police

IPV, intimate partner violence.

The study involved 10 IPV survivors aged between 30 and 50 years who were counselled and supported by church leaders in two provinces of South Africa. Nine participants were married over 9–27 years, and one was a divorcee of 5 years. The participants experienced abuse over a period of 3–30 years.

### Survivors revealed experiences of various forms of intimate partner violence: Theme 1

Various forms of IPV were encountered by survivors. Five categories emerged from this first theme: physical injury, psychological distress, financial distress, sexual assault, and social isolation.

#### Category 1.1: Psychological violence

Participants were psychologically affected because of verbal attacks, death threats and partners’ infidelities, which led to suicidal ideation and attempted suicide. Three participants explained their ordeal as follows:

‘It is very difficult to live with someone who abuses you emotionally by having repeated extra-marital relationships, which also resulted in children. I caught him with two of my helpers …’ (Province 1, Participant F4, 38 years)

‘I was afraid because he even threatened me and said if I have got that thing, I should have shot you. By that thing, he meant the gun. He didn’t mention it in words … he can even come up with a newspaper article where a certain woman was burnt by hot water and said that if someone is not complying, this is what happens. If, for example, if on the radio, they report about a husband who has killed a wife with the children, he would say yes, that is a brave man, sometimes things need to end like that.’ (Province 2, Participant F7, 54 years)

‘There was a time when I couldn’t take it … I think twice that I tried to commit suicide because of what was happening. The last time I tried to commit suicide, I remember he even told me don’t make your problem my problem. I had taken a lot of pills.’ (Province 1, Participant F5, 34 years)

#### Category 1.2: Physical violence

More than half of the participants were beaten up, kicked around, slapped on their faces and even strangled and two experienced pregnancy complications. Two participants confirmed physical violence and said:

‘There was a time when he would beat me, and I had a black eye; I had to wear heavy eye shadow and put on sunglasses because that Sunday, I was to be the master of ceremonies, and I couldn’t cancel. My mouth was also swollen; I had to say I had a tooth problem and just covered my lips with a face cloth since I was standing in front of people … even when he would beat me up the previous night, the next morning on a Sunday we would go to church together, we would dress up and wear matching clothes to please the pastors and show that all is well.’ (Province 2, Participant F6, 36 years)

‘He used to beat me up while I was pregnant with this child he didn’t want. He would hit me with his fists, and mostly he would hit me on my belly, saying I don’t want this child … He would hit me and even strangle me. He would also hit me on the face, ribs and back. With all these assaults, I will be expected to behave as if nothing happened at church.’ (Province 2, Participant F8, 37 years)

In line with these findings, Lutgendorf (2019:473) asserts that pregnancy did not exempt women from abuse.

#### Category 1.3: Financial abuse

Participants were financially abused because of irresponsible husbands, expecting most bills to be paid by their wives, sometimes because of disparities in earnings. One participant who was financially controlled and exploited said:

‘He depended on me as he was not working … he was trying to … as self-employed. The capital would come from me, and he expected it to be endless. You will give him capital, and you don’t see any profit, and nothing will come back to you. You will be pumping the money, and nobody is going to pay you back or share the profit. Another thing was that as he became more abusive, I thought maybe if I give him some money, all the salary … this thing will be okay, and it will be okay for maybe only three days; thereafter, we go back again to the abusive behaviour.’ (Province 2, Participant F7, 54 years)

#### Category 1.4: Sexual abuse

Some participants experienced sexual abuse. They also could not negotiate condom use and were forced to participate in sexual activities. To confirm sexual abuse, one participant experienced repeated sexually transmitted infections (STIs) and expressed the following:

‘He insisted that condoms are not used in his house, and when I told him I wanted to protect myself from sexually transmitted infections, he would still blame me and tell me I knew where I got the infection. I ended up going to the clinic when it was bad, and I was feeling a lot of pain, and I couldn’t stay anymore when I had discharges and abscesses, knowing fully well that I was faithful. He forced me to have sex with him. It was unbearable knowing that I always had infections.’ (Province 2, Participant F6, 36 years)

‘He would refuse to use the condoms; and he would tell me he would not come back home again. My problem is that I don’t know his status, and I worry about the way he conducts himself.’ (Province 2, Participant F8, 37 years)

#### Category 1.5: Social isolation

Survivors were socially isolated from family and friends, being manipulated to stay away willingly or unwillingly from family and friends. One participant confirmed social isolation as follows:

‘Yes, I’m not even allowed to communicate with others on my phone. If it rings and he is there, it is a problem. He would ask whom I am communicating with, or he would take it while I’m busy using it. He used to ask his sister to check on my phone the calls that were received and dialled so that she can send the numbers to him, then he would call all those numbers to find out who those people were.’ (Province 2, Participant F6, 36 years)

‘He will not allow you to have company with a certain person. He will choose your friends; he will choose where you go; he will choose your clothes; he will choose your ringtone; he will choose your … whatever is being chosen for you.’ (Province 2, Participant F7, 54 years)

### Survivors encountered hardships while looking for assistance after intimate partner violence: Theme 2

Reporting IPV to family, church leaders and the police was found to be a serious challenge for survivors. Participants feared the potential stigmatisation from their family, church leaders and members (Crowe, Overstreet & Murray 2021:7475). This situation was considered traumatic to the survivors as having one’s own family criticise and condemn their action of disclosure. Church leaders were seen as lacking knowledge on the prevention of abuse and not offering any effective support as this would contradict their inherent support for patriarchy. Furthermore, police officers were viewed as being biased and often blamed IPV survivors for instigating the violence they had experienced (Gumani 2022:380).

#### Category 2.1: Difficulties when disclosing partner abuse to their family

Participants experienced challenges because of dissatisfaction with family, religious and cultural expectations. Two participants confirmed that their parents and in-laws never addressed the issue:

‘I would tell my mother-in-law, knowing that she is a pastor. She counsels married couples, so I trusted she would know how to handle the situation. Furthermore, the actual abuse was not addressed, or the actual problem was not addressed. It was always going back to addressing the wife. You are the wife … your role is to … So it always felt like instead of being a remedy, it became more of you should rather not do this so that it should never happen again.’ (Province 1, Participant F2, 35 years)

‘I grew up in a family that is too traditional, they don’t believe in divorce. And they also call themselves Christians. So irrespective of the challenges that you are going through, they talk you into staying in the marriage.’ (Province 1, Participant F3, 35 years)

In line with these findings, women are said to experience further abuse by family members and in-laws as there was little consideration for the women during dispute resolution. Furthermore, in-laws are said to be biased and did very little to end the abuse (Mogstad, Dryding & Fiorotto 2016:8).

#### Category 2.2: Difficulties when disclosing partner abuse to their church leaders

Participants who encountered problems disclosing IPV to their pastors became frustrated because they did not get the support they needed through counselling. Interestingly, this study found that survivors and their church leaders were not keen to choose divorce as an alternative to prevent IPV:

‘It’s all the same. The pastors are just looking for your money, and when they finish you, they will abandon you. They take you like an orange, squeeze the juice out and throw it away. All these church services I attended, sacrificing everything to find myself in the house of God. I thought when I was having my marriage problems and divorce, it would be church people who would be closer to me, only to find that they would be the ones who would push me away. The elders of the church would also run away from me as I came nearer; it meant that the lady pastor told the elders that she didn’t trust me any longer. I didn’t find help; I ended up not going to church.’ (Province 2, Participant F6, 36 years)

‘Even pastors tell me not to get into divorce; they told me God will settle everything.’ (Province 2, Participant F8, 37 years)

#### Category 2.3: Difficulties when disclosing partner abuse to police

Some participants reported facing challenges when seeking help from the South African Police Service (SAPS). These challenges at local police stations confirmed participants’ frustrations:

‘No, the police just came and told him to stop beating a woman and all that. Nothing happened. They didn’t arrest him.’ (Province 1, Participant F1, 30 years)

‘You see, when you get to the police station at the counter, you know the police are always asking you in front of everyone what your problem is? You think maybe I’m just going to go there and find it’s only men, and maybe I’m not going to find women who will understand.’ (Province 1, Participant F2, 35 years)

## Discussions

The findings of this study showed different types of abuse experienced by IPV survivors in the selected Pentecostal churches in South Africa. Their experiences were congruent with Kadir Shahar et al.’s (2020:1) assertion that women are being abused in different ways. The study also found that most participants overlook their abuse for various reasons, including protecting the children, maintaining the peace, religious obligation, or just being in favour of staying married to avoid the stigma attached to divorce. Religious commitment to remain married was a dominant motivator for women to remain in abusive relationships. Women feared being stigmatised by their families and church members (Crowe et al. 2021:7475).

It was also found that women consider some forms of abuse more tolerable than others. For instance, in this study, participants mainly reported physical violence to the police because psychological, including financial violence, is considered tolerable and less violent even though there is evidence of their damning effects. This study contrasts with Mtaita et al.’s (2021:4) assertion that psychological abuse is considered unbearable while physical abuse was accepted by many participants as a ‘normal behaviour’.

The study uncovered some interesting disparities concerning how the two participant groups (urban and rural) experienced IPV. Both groups experienced emotional abuse, with some expressing suicidal ideation and attempted suicide; this was more prominent among the urban cohort; conversely, the rural cohort received death threats. In terms of financial abuse, the rural cohort was mostly financially dependent on their partners when compared with their urban counterpart, who was found to be the most financially stable and was assigned more financial responsibilities and consequently taken advantage of. This is congruent with the study by Mokwena and Maake (2022:20118, 20126, 20127) on poverty being a contributor to women’s experience of IPV as well as women of a better financial standing also being found to suffer at the hands of their partners (Fapohunda et al. 2021:655). This reveals that neither cohort was found to be immune from abuse because of their socio-economic status. Interestingly, Malesa (2021:155) found that only women who are breadwinners are afforded equal status.

While there was no evidence to show that rural women suffered more sexual violence because of their location, urban women, however, were found to be probable sufferers of emotional violence in comparison to their rural counterparts (Fapohunda et al. 2021:656, 657). This is congruent with the findings of this study on episodes of suicidal ideation and attempted suicide among the urban cohort.

Another prominent issue was that of marital rape. Marital rape is commonly accepted by family and church leaders. This is in line with Selepe, Lindegger and Govender (2021:6); Mkhize et al. (2022:8), that marital rape is considered justifiable and rightfully deserved by husbands: a concept in both patriarchal and religious practices that promote female passivity, making it difficult for marital rape to be acknowledged as IPV. As stated earlier, Mkhize et al. (2022:8) posited that the view that couples should submit to one another, including their bodies, is often practised one-sided, as this submission only benefits males. Marital rape was found to be more accepted within rural contexts. Urban women considered this act to violate their rights, while rural women were more disadvantaged because of adherence to culture and religion.

Intimate partner violence was found to have adverse health effects on survivors. Some women experienced injuries from physical violence, pregnancy complications, repeated STIs from cheating partners and susceptibility to HIV because of women’s inability to negotiate condom use (Willie et al. 2020:6). The study found that little attention was given to the seriousness of these adverse effects. There was stigma and shame surrounding abuse. This led to some women accepting their circumstances to protect their marital status, family and image. Many survivors seemingly did not seek medical attention for fear of further stigmatisation. This is congruent with Crowe et al.’s (2021:7457) assertion that even professionals might stigmatise IPV survivors to an extent where they are discouraged from seeking help.

The study found that survivors suffered the consequences of psychological trauma. This is congruent with Chandan et al.’s (2020:562) study, asserting that IPV is linked to ‘poor mental health outcomes’ leading to disorders such as anxiety, depression, and suicidal thoughts. Consequently, the longer women remain in abusive relationships, the more vulnerable they are to various health risks.

The study also focused on how women felt about the experience and outcome of their disclosure of abuse to their families, pastors, and law enforcement officials. Furthermore, the study found that family and church leaders opposed divorce, which influenced the counselling and support women received. Survivors’ experience of church leaders opposing divorce is in line with Le Roux and Bowers-Du Toit (2017:22 -23) as well as Khosa-Nkatini and Mofokeng’s (2023:5) assertion that churches put more effort towards preserving the sanctity of marriage. Likewise, participants felt that church leaders were not so keen in ending abuse but encouraging them to stay in the same abusive environment.

In this study, survivors viewed some police officers as insensitive and harsh when abuse incidents were reported to them. Police were found to not take the participants’ plight seriously. This is congruent with the study of (Yesufu 2022:97), who identified police as lacking the capacity and sensitivity required to address IPV matters, and that makes victims feel responsible or at fault, thus contributing to secondary victimisation (Gouws 2022:2; Gumani 2022:380).

Some families were exasperated with women’s experience of abuse, while other women felt the abuse was aggravated by the reporting, especially after reporting to the police. It was also evident, however, that survivors who disclosed their abuse to the police never wanted their partners to be arrested but wished that their partners would stop abusing them after police involvement. Unfortunately, there exists a gap between the police and church leaders. A formal link between the two partners through church leaders’ involvement with community policing forums and other GBV platforms could motivate victims of IPV to freely report their cases to the police.

Based on the given discussions, some guidelines are offered as corrective action to address the church’s response to IPV. It has been found that church leaders have much potential to be instrumental in the prevention of IPV and can become key figures in the support of survivors by:

- acknowledging the plight of survivors as a result of IPV and taking corrective action to address it;
- using the pulpit to address issues of silence and renouncing the notion that IPV is a family matter;
- advising survivors that all forms of abuse, bearable or unbearable, should not be taken for granted or overlooked;
- abstaining from encouraging survivors to endure IPV or compelling survivors to remain in abusive relationships for the sanctity of marriage;
- discouraging family and the church from blaming and stigmatising survivors who choose to disclose their abuse or divorce their partners;
- encouraging survivors to disclose abuse to their families, pastors, medical professionals, law enforcement officials, as well as mental healthcare professionals;
- encouraging men to refrain from abusing their spouses;
- encouraging perpetrators to seek professional help and advising them to attend perpetrator programmes;
- addressing issues of marital rape by helping husbands to unlearn acceptance of marital rape and acknowledging that marital rape is a form of sexual abuse;
- participating in community forums and other GBV platforms could motivate victims of IPV to freely report their cases to the police;
- linking with police officers and other health professionals to address issues of secondary victimisation.

## Limitations

Generalisability was the study’s major limitation as the researchers explored IPV experiences in only two provinces, Gauteng and Limpopo, out of the nine provinces. It also only concerned itself with Pentecostal churches, even though churches across the board are struggling with issues of IPV. Furthermore, while the study consisted of participants in metropolitan and non-urban areas, a comprehensive comparison between the two was not carried out. The study concerned itself with only the experiences of selected IPV survivors.

## Conclusion

The findings reveal a sense of helplessness experienced by IPV survivors because family, leaders in the church and the police failed them regarding support. Church leaders were blamed for not being well-versed in providing counselling on IPV. This status quo played a significant role in participants’ experiences. It was also apparent that IPV’s effects on survivors’ mental health were evidenced by the severe emotional stress that led to some survivors having suicidal ideation and repeated suicide attempts. Suicidal ideation was found to be concerning in that it led to suicide attempts.

Furthermore, cultural and religious beliefs played a significant role. More research is recommended for studying the type of support that survivors require after disclosing IPV to stakeholders such as family, church leaders and the police and exploring their needs in terms of inclusive support. There is also a need to explore the views of community health nurses and other aforementioned stakeholders on providing holistic assistance. The recommendation for nursing practice is that community health nurses partner with church leaders to fight the scourge of IPV because most Christian victims and survivors of IPV prefer to seek religious counselling.

For church leaders to be effective in preventing IPV, they should be in partnership with other stakeholders in the community, including community health nurses. They should forms teams that meet regularly to address key strategies to prevent IPV, strategies that address societal and religious norms that promote violence as well as formulating prevention and perpetrator programmes.
